# 31 Establishing Pressure Parameters and Viability of a 3D Printed Dynamic Microstomia Prevention Orthosis

**DOI:** 10.1093/jbcr/irae036.031

**Published:** 2024-04-17

**Authors:** Christopher Whitehead, Courtney Hall

**Affiliations:** Shriners Children's Texas, Galveston, Texas; Shriner's, Galveston, Texas; Shriners Children's Texas, Galveston, Texas; Shriner's, Galveston, Texas

## Abstract

**Introduction:**

Microstomia prevention orthoses (MPOs) are a standard of care for perioral burns to prevent and correct contractures due to hypertrophic burn scar. A simple, cost effective 3D printed MPO was designed with a dynamic middle component and two mouthpieces. Our goal was to establish parameters for providing appropriate pressure and to examine the viability of the design before implementation. Although effective pressure standards for dynamic MPOs have not been established, our objective is 15-25 mmHg based on the current literature for effective pressure therapy of hypertrophic burn scars.

**Methods:**

Multiple MPOs were fabricated with varying levels of elasticity. Two variables were modified: the length in 1 cm increments (9 cm to 13 cm) and the level of infill by 10% (80% to 100%), resulting in fifteen separate devices. A digital force gauge was used to measure peak force in four positions of compression to determine pressure for the MPOs along the varying levels of mouth opening. Starting with the mouth pieces 1 cm apart, the distance was increased by 1 cm increments (1 cm to 4 cm) and measured. Using the force divided by the surface area of the device, the pressure was calculated. Three of the MPOs that provided consistent pressure through the widest coverage of mouth opening were selected for further testing. Ten duplicates of each of the three MPOs were fabricated and re-tested in the same four positions to assess the precision of the design. Another ten duplicates of each size were measured for force at one-minute intervals for ten minutes with the mouthpieces 2.5 cm apart to assess a decline in elasticity over time.

**Results:**

The lengths of 10 cm, 11 cm, and 12 cm with 80% infill were selected because they provide coverage from 27.3 mmHg at 1 cm to 21.2 mmHg at 4 cm apart. The mean, standard deviation, and coefficient of variation were calculated to assess precision for all sizes in all four positions. The standard deviation ranged from 0.21 to 0.49 mmHg and was 0.9 % to 2.2% of the mean. When assessing decline in elasticity, the MPOs dropped an average of 3.97 mmHg over a 10-minute period with 76.97% of the decrease occurring within the first three minutes.

**Conclusions:**

The use of three MPOs of the same infill but different lengths provide sufficient pressure across the various degrees of mouth opening. They demonstrate consistent pressures between orthoses of the same length and infill. While there is an initial drop in pressure after application, the devices continue to provide therapeutic levels of pressure during use.

**Applicability of Research to Practice:**

These orthoses have been shown to reliably provide pressure and are ready to be incorporated into clinical practice. A sizing chart can be used to select the correct size MPO based on horizontal mouth opening. This simple but effective design has the potential to provide a low cost solution for burn microstomia with worldwide availability.

